# Diffusion-weighted imaging in differentiating mid-course responders to chemotherapy for long-bone osteosarcoma compared to the histologic response: an update

**DOI:** 10.1007/s00247-021-05037-4

**Published:** 2021-04-20

**Authors:** Céline Habre, Alexia Dabadie, Anderson D. Loundou, Jean-Bruno Banos, Catherine Desvignes, Harmony Pico, Audrey Aschero, Nathalie Colavolpe, Charlotte Seiler, Corinne Bouvier, Emilie Peltier, Jean-Claude Gentet, Christiane Baunin, Pascal Auquier, Philippe Petit

**Affiliations:** 1 Division of Pediatric Radiology, Hôpital Timone Enfants, Assistance publique - Hôpitaux de Marseille, 264 Rue Sainte Pierre, 13385 Marseille Cedex 05, France; 2 Division of Pediatric Onco-Hematology, Hôpitaux Universitaires de Genève, Genève, Suisse; 3 Division of Statistics and Methodology for Clinical Research, Assistance publique - Hôpitaux de Marseille, Aix-Marseille Université, Marseille, France; 4 Anatomopathology Laboratory, Hôpital Timone Enfants, Assistance publique - Hôpitaux de Marseille, Marseille, France; 5 Division of Pediatric Radiology and Prenatal Imaging, Hôpital Timone Enfants, Assistance publique - Hôpitaux de Marseille, Marseille, France; 6 Division of Pediatric Orthopedic Surgery, Hôpital Timone Enfants, Assistance publique - Hôpitaux de Marseille, Marseille, France

**Keywords:** Bone neoplasm, Children, Diffusion-weighted imaging, Magnetic resonance imaging, Osteosarcoma, Therapeutic response

## Abstract

**Background:**

Diffusion-weighted imaging (DWI) has been described to correlate with tumoural necrosis in response to preoperative chemotherapy for osteosarcoma.

**Objective:**

To assess the accuracy of DWI in evaluating the response to neoadjuvant chemotherapy at the mid-course treatment of long-bone osteosarcoma and in predicting survival.

**Materials and methods:**

We conducted a prospective single-centre study over a continuous period of 11 years. Consecutive patients younger than 20 years treated with a neoadjuvant regimen for peripheral conventional osteosarcoma were eligible for inclusion. Magnetic resonance imaging (MRI) with DWI was performed at diagnosis, and mid- and end-course chemotherapy with mean apparent diffusion coefficients (ADC) calculated at each time point. A percentage less than or equal to 10% of the viable residual tissue at the histological analysis of the surgical specimen was defined as a good responder to chemotherapy. Survival comparisons were calculated using the Kaplan-Meier method. Uni- and multivariate analyses with ADC change were performed by Cox modelling. This is an expansion and update of our previous work.

**Results:**

Twenty-six patients between the ages of 4.8 and 19.6 years were included, of whom 14 were good responders. At mid-course chemotherapy, good responders had significantly higher mean ADC values (*P*=0.046) and a higher increase in ADC (*P*=0.015) than poor responders. The ADC change from diagnosis to mid-course MRI did not appear to be a prognosticator of survival and did not impact survival rates of both groups.

**Conclusion:**

DWI at mid-course preoperative chemotherapy for osteosarcoma should be considered to evaluate the degree of histological necrosis and to predict survival. The anticipation of a response to neoadjuvant treatment by DWI may have potential implications on preoperative management.

## Introduction

Osteosarcoma is the most common malignant bone tumour in adolescents and young adults [1]. Neoadjuvant chemotherapy has improved survival in localized osteosarcoma by downstaging the T (tumour) stage of the tumour before surgical resection [2]. The response to preoperative chemotherapy is a strong predictor of local recurrence and outcome, and impacts decisions regarding postoperative checmotherapy and further surgery [3, 4].

Postoperative histological analysis of the bone specimen remains the reference standard to determine the response to neoadjuvant chemotherapy [5–7]. Ideally, the assessment of the effectiveness of chemotherapy and prognostic stratification would be done preoperatively.

Magnetic resonance imaging (MRI) is part of the routine management of osteosarcoma for diagnosis, extension work-up and surgical planning [4, 8]. Thus far, neoadjuvant regimen adjustment decisions have been based on visual interpretation of volume change during neoadjuvant chemotherapy. Unfortunately, this does not provide reliable predictive values of tumour behaviour [8–10].

Diffusion-weighted imaging (DWI) has been used to characterize various bone malignancies and to monitor their response to treatment [11]. Several authors have evaluated the ability of DWI to predict osteosarcoma response to neoadjuvant chemotherapy in clinical practice [12–15]. However, these studies have only compared apparent diffusion coefficient (ADC) values of osteosarcoma recorded before treatment to those obtained after the completion of chemotherapy and immediately before surgery. In our opinion, this information was obtained too late during the course of neoadjuvant treatment to allow any modification of potentially unsuccessful treatment.

We have previously demonstrated promising results regarding mean ADC differential measurement between induction and mid-course neoadjuvant treatment evaluation to identify poor histological responders [16]. The ADC differential between these two time points enabled us to detect 57% of the poor responders with 100% specificity. However, our study suffered from a small sample size. We have continued to study patients to increase our cohort to validate our preliminary finding.

Our main objective was to test the ability of DWI to assess early tumour response to neoadjuvant chemotherapy by comparing tumoural ADC values measured at mid-course regimen with the histological assessment of response after completion of preoperative chemotherapy and surgery. Our secondary objective was to determine tumoural ADC values at the end of chemotherapy. Our third objective was to test whether DWI could serve as a prognostic factor of survival in comparison to the reference standard and other known prognosticators.

## Materials and methods

### Study design

We conducted a prospective monocentric study on a consecutive series of children and adolescents with long-bone osteosarcoma treated at our institution since 2005. This study was approved by our institutional review board and ethics committee.

### Participants

The enrollment period spanned from 2005 to 2016. Inclusion criteria were patients younger than 20 years old with a diagnosis of long-bone conventional osteosarcoma, localized or initially metastatic, treated by neoadjuvant regimen, and undergoing MRI at diagnosis (MRI-1), and mid-course (MRI-2) and end-course (MRI-3) chemotherapy. We excluded patients with telangiectatic osteosarcoma because of their mainly cystic matrix.

All patients were treated according to the French 2006 osteosarcoma treatment protocol [4], which corresponds to seven courses of high-dose methotrexate (12 g/m^2^) and two courses of ifosfamide/etoposide for 4 days (3 g/m^2^ and 75 mg/m^2^, respectively) administered over 13 weeks.

Eligible participants were identified at the time of radiologic diagnosis with enrollment made at imaging work-up. Written informed consent was obtained from patients and parents before the initial MR examinations.

### Magnetic resonance imaging

Three MRI examinations were planned: the first one (MRI-1) at baseline before surgical biopsy, the second one (MRI-2) halfway through chemotherapy (i.e. at week 7), and the third one (MRI-3) at the end of neoadjuvant chemotherapy (i.e. at week 14, immediately before surgery).

All examinations were performed on the same 1.5-tesla (T) MRI unit (Achieva; Philips, Bests, The Netherlands) without sedation and for a maximal duration of 45 min.

The protocol included the following conventional pre-contrast images:

- Body coil for joint-to-joint coverage (for skip metastasis depiction), sagittal and coronal plane short tau inversion recovery (STIR) (repetition time [TR]>2,500 ms/echo time [TE] 70 ms/inversion time [TI] 140 ms, matrix 320×256, field of view [FOV] 500 mm, 3-mm slice thickness)
- Same surface coil centered on the bone tumour, with FOV ranging from 200 to 240 mm, 3-mm slice thickness:
- T1 coronal spin echo (435 ms/TE 18 ms, matrix 304×242)
- T2 axial turbo spin echo (TR 5,400 ms/TE 110 ms, matrix 197×400)
- T2 sagittal fat-suppressed turbo spin echo (4,504 ms/TE 52 ms, matrix 208×165).

### Contrast-enhanced images

Post-contrast sequences were obtained after gadolinium injection of 0.1 mmol/kg body weight gadoteric acid (Dotarem; Guerbet, Roissy, France) using the following protocol:

- T1 gradient echo (GE) dynamic with subtraction (TR 11 ms/TE 4.2 ms, matrix 256×163, 15 dynamic scans, dynamic scan time 17 s)
- Three-dimensional (3-D) T1 GE with spectral fat suppression after gadolinium injection (TR 14 ms/TE 6.9 ms, matrix 256×114).

### Diffusion-weighted imaging

DWI was obtained in the coronal plane before contrast injection using a turbo spin echo (TSE) diffusion-weighted sequence (TR 1,500 ms/TE 138 ms, matrix 112×89, TSE factor 16), acquired along three gradient directions and four b values (0, 300, 600 and 900 s/mm^2^). This sequence provided one slice of 20-mm thickness in the long axis of the bone in 1.46 min, parallel to the plane in which the future histological specimen was to be analysed.

### Image analysis

**Fig. 1:**
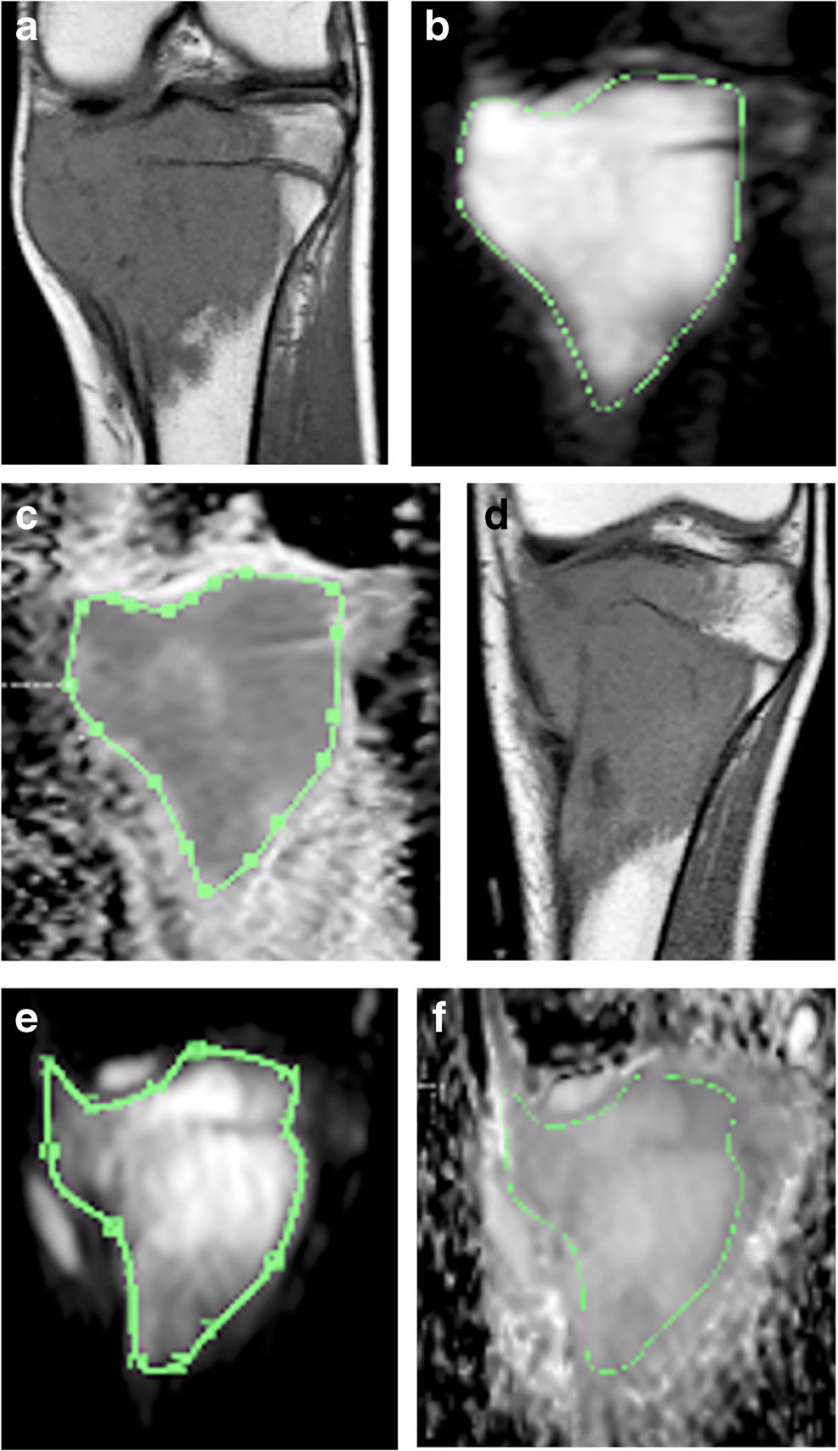
Osteosarcoma of the proximal tibial metaphysis with extension to physis, epiphysis and joint surface in a 9.5-year-old boy (patient 17). **a−f** Coronal planes acquired at T1-W (**a, d**), DWI at b0 value (**b, e**) and corresponding apparent diffusion coefficient (ADC) maps (**c, f**). Examples of ADC calculation (good responder) at MRI-1 (**a, b, c**) and MRI-2 (**d, e, f**): a region of interest is manually drawn around the tumour along its longer axis to calculate the mean ADC of the tumour. A qualitative assessment of the ADC map shows a decrease in signal intensity at mid-course chemotherapy

Image analysis was performed with a Philips View Forum processing console (Philips Healthcare). DWI and conventional sequences were analysed concomitantly with the screen divided into four quadrants dedicated to the four following coronal sequences: DWI obtained at b=0, ADC map, precontrast T1-W and post-contrast fat-suppressed T1-W. We chose a median coronal plane through the greatest axis of the tumour to closely reproduce the section of the surgical specimen to used by the pathologist. Because we did not use the same FOV, gap and slice thickness for both pre- and post-contrast T1-W and DWI sequences, we could not copy and paste the tumour limits delineated on one sequence to another. However, using the same magnification and anatomical landmarks on the screen on both T1-W and DWI sequences, manual segmentation of the osteosarcoma contour was feasible on the b0 image. This region of interest (ROI) was then dragged to the ADC map from which the average ADC value of the tumor was derived (Fig. 1).

ADC was calculated using the usual formula: ADC=(ln S0×S900)/(b900−b0) (where S0 is the signal intensity if b=0 and S900 is the signal intensity if b=900, and ln=natural logarithm), expressed in mm^2^/s.

Three ADC values were recorded, ADC1, ADC2 and ADC3 (for MRI-1, MRI-2 and MRI-3, respectively), from which four other parameters were calculated, ADC absolute differential between first and second MRI (ADC2−ADC1), ADC absolute differential between first and third MRI (ADC3−ADC1), ADC relative differential expressed as a percentage between first and second MRI, defined as the ratio ([ADC2−ADC1/ADC1]×100), ADC relative differential between first and third MRI ([ADC3−ADC1/ADC1]×100).

Because our previous study [16] showed no significant intra- and interobserver variability, the measurements were made by one senior radiologist (P.P.), who was blinded to patient clinical information and to the final histological diagnosis.

### Histology reference standard

After surgical resection, bone specimens were sent for histological analysis according to Huvos’ grading system [5, 6].

The percentage of viable residual cells was calculated from a 5-mm coronal slice of the specimen along the greatest axis of the tumour, including soft-tissue extension. A good response to neoadjuvant chemotherapy was defined as a surgical specimen section composed of 10% or less of viable tumoural cells, and a poor response as more than 10% of viable tumoural cells.

### Survival end points

Overall survival was defined as the time from diagnosis to the last follow-up for patients in complete remission or to death from any cause. Event-free survival was defined as the time from the date of diagnosis to the date of first progression, either local relapse or metastasis. Overall survival and event-free survival were established for good and poor histological responders according to ADC measurements. We selected known risk factors reported in the literature, i.e. tumour volume, metastases at diagnosis, and poor histological response to neoadjuvant chemotherapy, as well as ADC change from MRI-1 to MRI-2, to test their prognostic potential for overall survival.

### Statistical analysis

Descriptive statistics were used to describe population characteristics. A comparison of average ADC values and their absolute and relative differentials for the good responder versus poor responder groups was performed using nonparametric Mann-Whitney tests. We carried out a receiver operating characteristic curve analysis to assess the performance of the three parameters to discriminate between good and poor responders: average ADC, ADC absolute and relative differentials. For each parameter, we chose the cutoff identifying the best sensitivity for a 100% specificity.

Overall survival and event-free survival were calculated for both ADC change and the histological response using the Kaplan-Meier method and log-rank test. Uni- and multivariate analyses using Cox modelling were done for potential prognosticators of overall survival.

We did not calculate the sample size.

For all tests, a *P*-value of 0.05 was considered the threshold for significance. Statistical analysis was done using IBM SPSS statistics version 20 (SPSS, Chicago, IL).

Three missing ADC measurements at MRI-3 were handled by excluding these subjects from the analysis of ADC after chemotherapy. One patient with missing data on the reference standard test was also excluded.

## Results

### Participants

**Fig. 2:**
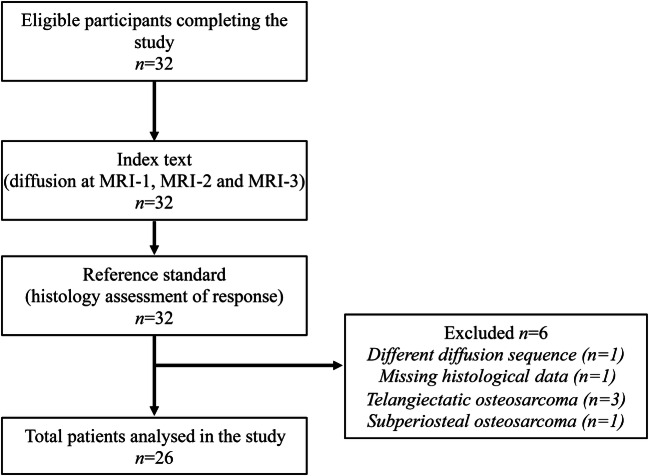
Flow diagram of participants

From 2005 to 2016, 32 patients were assessed for initial eligibility and completed the study. Six patients were excluded from the analysis because of an absent index test in one (i.e. a different diffusion sequence had been acquired), missing data on histological analysis in one, biopsy-proven telangiectatic osteosarcoma in three and periosteal osteosarcoma in one. In total, 26 patients were analysed. Figure 2 shows the flow of participants through the study.

The age of patients ranged from 4.7 to 19.6 years (median: 13 years), with a male:female ratio of 11:15. Tumours were localized in decreasing order to the distal femur (14/26, 54%), proximal tibia (8/26, 31%), femoral diaphysis (2/26, 8%) and proximal humerus (2/26, 8%).

Histology demonstrated 14 good responders (54%) and 12 poor responders to chemotherapy (46%) with a similar distribution between the subtypes of conventional osteosarcomas. Among the 26 conventional osteosarcomas, there were 20 osteoblastic (77%), 3 chondroblastic (12%) and 3 mixed osteoblastic/chondroblastic (12%). Tumour volume ranged from 123 to 405 cm^3^ (average: 240 cm^3^). Metastases were present at diagnostic in 8/26 (31%) patients. Surgical margins were negative in all subjects. One patient had an inherited cancer predisposition syndrome (Li Fraumeni syndrome, patient number 20).

Table 1 provides patients’ characteristics.

**Table 1:** Patients characteristics

Patient	Age (years)	Gender	Predisposing condition	Location	Tumour volume (cm^3^)	Metastasis at presentation	Osteosarcoma subtype	Residual tumor cells (%)	Histological response to chemotherapy
1	18.5	F	–	distal femur	60.7	–	osteoblastic	8	Good
2	15	M	–	distal femur	1,199.5	–	osteochondroblastic	7	Good
3	14	M	–	distal femur	405.2	–	chondroblastic	1	Good
4	12.2	M	–	proximal humerus	101.6	–	chondroblastic	2	Good
5	19.6	F	–	proximal tibia	107.3	–	osteoblastic	16	Poor
6	16.3	F	–	distal femur	123	–	osteoblastic	5	Good
7	14.4	F	–	distal femur	221.4	–	osteochondroblastic	20	Poor
8	10.5	F	–	proximal tibia	61.7	lung	osteoblastic	75	Poor
9	4.7	M	–	distal femur	98.4	lung	osteoblastic	70	Poor
10	12.7	F	–	distal femur	46.1	–	osteoblastic	13	Poor
11	8.8	M	–	distal femur	94.2	–	osteoblastic	8	Good
12	16.5	M	–	proximal tibia	147.1	lung	osteoblastic	15	Poor
13	16.4	F	–	femoral diaphysis	320.4	–	osteoblastic	1	Good
14	14.4	F	–	distal femur	179	–	osteochondroblastic	20	Poor
15	16.3	F	–	proximal tibia	227	–	osteoblastic	20	Poor
16	8.7	F	–	distal femur	368.1	bone	osteoblastic	12	Poor
17	9.5	M	–	proximal tibia	181.4	lung	osteoblastic	3	Good
18	12.7	F	–	distal femur	264	lung	osteoblastic	23	Poor
19	9.4	F	–	distal femur	437.9	bone	osteoblastic	1	Good
20	8.9	F	Li Fraumeni	distal femur	64.7	–	osteoblastic	10	Good
21	11.8	M	–	proximal tibia	405	lung	chondroblastic	25	Poor
22	7.5	M	–	femoral diaphysis	105.9	–	osteoblastic	0	Good
23	14.6	M	–	distal femur	323.7	–	osteoblastic	25	Poor
24	16.9	M	–	proximal tibia	251.5	–	osteoblastic	9	Good
25	10	F	–	proximal tibia	70.1	–	osteoblastic	7	Good
26	13.3	F	–	proximal humerus	456.8	–	osteoblastic	3	Good

High-risk factors for poor outcome include tumour size, initial metastases, poor histological response and unresectable primary tumour. Because surgical margins were negative for all patients, this feature is ommtted from the table. *F* female, *M* male

### Test results

Table 2 shows ADC values for each patient at MRI-1, MRI-2 and MRI-3, their absolute and relative differentials and their corresponding histology. Mean ADC value increased along with the three time points in both good and poor responder groups.

**Table 2:** Apparent diffusion coefficient (ADC) values compared to histological assessment of tumoural response to neoadjuvant chemotherapy in each patient

Patient	ADC1	ADC2	ADC3	ADC2–ADC1	(ADC2–ADC1) /ADC1 × 100	ADC3–ADC1	(ADC3–ADC1) /ADC1 × 100	Histology
1	1.32	1.97	1.9	0.65	0.49	0.58	0.44	Good
2	1.75	2.15	2.37	0.4	0.23	0.62	0.35	Good
3	1.36	1.7	1.78	0.34	0.25	0.42	0.31	Good
4	1.47	1.86	–	0.39	0.27	–	–	Good
5	1.47	1.39	2.13	−0.08	−0.05	0.66	0.45	Poor
6	1.38	2.07	1.87	0.69	0.50	0.49	0.36	Good
7	2.02	1.73	1.7	−0.29	−0.14	−0.32	−0.16	Poor
8	1.42	1.71	1.62	0.29	0.20	0.2	0.14	Poor
9	1.39	1.84	1.88	0.45	0.32	0.49	0.35	Poor
10	1.55	1.78	2.05	0.23	0.15	0.5	0.32	Poor
11	1.37	2.21	2.67	0.84	0.61	1.3	0.95	Good
12	1.56	1.93	2.24	0.37	0.24	0.68	0.44	Poor
13	2.14	2.85	2.87	0.71	0.33	0.73	0.34	Good
14	1.41	2	2.07	0.59	0.42	0.66	0.47	Poor
15	1.38	1.7	1.74	0.32	0.23	0.36	0.26	Poor
16	1.46	2.15	1.52	0.69	0.47	0.06	0.04	Poor
17	1.24	2.05	2.41	0.81	0.65	1.17	0.94	Good
18	1.3	1.23	1.74	−0.07	−0.05	0.44	0.34	Poor
19	1.93	2.06	1.85	0.13	0.07	−0.08	−0.04	Good
20	1.49	1.87	2.46	038	0.26	0.97	0.65	Good
21	1.65	1.89	1.79	0.24	0.15	0.14	0.08	Poor
22	1.93	2.02	–	0.09	0.05	–	–	Good
23	1.5	1.85	1.77	0.35	0.23	0.27	0.18	Poor
24	1.22	1.85	1.95	0.63	0.52	0.73	0.60	Good
25	1.02	1.66	1.81	0.64	0.63	0.79	0.77	Good
26	1.27	1.76	2.23	0.49	0.39	0.96	0.76	Good

At mid-course chemotherapy, the mean ADC value change was significantly higher among the good responders compared to the poor responders *(P=*0.046)*.* The increase in mean ADC value from MRI-1 to MRI-2 was significantly higher for the good responders (*P=*0.015) (Table 3).

**Table 3:** Values (mean±standard deviation) at mid-course of neoadjuvant chemotherapy for good and poor responders

	Good responders (*n*=14)	Poor responders (*n*=12)	*P*-value^a^
ADC1	1.49±0.32	1.51±0.19	0.297
ADC2	2.00±0.29	1.77±0.25	0.046
ADC2−ADC1	0.51±0.23	0.26±0.28	0.015
(ADC2−ADC1)/ADC1	0.37±0.20	0.18±0.19	0.015

There is a significantly higher increase in the apparent diffusion coefficient (ADC) from baseline to mid-course treatment in the good responders group

^a^Mann-Whitney test

**Fig. 3:**
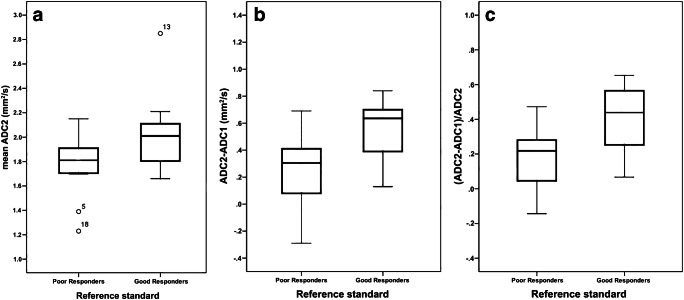
Box plots comparing apparent diffusion coefficient (ADC) values at MRI acquired at mid-course of chemotherapy (MRI-2). **a−c** Box plots compare ADC2 values (**a**), absolute differential ADC2−ADC1 values (**b**) and ratio (ADC2−ADC1)/ADC1×100 values (**c**). Good responders have higher mean ADC values and higher increases in ADC from initial to mid-course MRI than poor responders

The best discrimination between good versus poor responders at mid-course chemotherapy was obtained by the absolute differential ADC2−ADC1. An increment of 0.07 from ADC1 to ADC2 showed a 100% specificity to detect 25% of the poor responders (95% confidence interval [CI] 5.50–57.20%) with a diagnostic accuracy of 65.4% (95% CI 46.2–80.6%). Figure 3 provides box plots comparing the distribution of mean ADC values at mid-course chemotherapy and their differential to initial MRI between good and poor responders.

Mean ADC values, ADC absolute differentials and ADC relative differentials were significantly higher among the good responders after chemotherapy (Table 4).

**Table 4:** Values (mean±standard deviation) at the end of neoadjuvant chemotherapy for good and poor responders

	Good responders (*n*=14)	Poor responders (*n*=12)	*P*-value^a^
ADC1	1.49±0.32	1.51±0.19	0.297
ADC3	2.18±0.37	1.85±0.22	0.014
ADC3−ADC1	0.72±0.37	0.35±0.29	0.008
(ADC3−ADC1)/ADC1	0.54±0.29	0.24±0.19	0.012

There is a significantly higher increase in the apparent diffusion coefficient (ADC) from baseline to end-course treatment in the good responders group

^a^Mann-Whitney test

The good responders group, as defined by histology, demonstrated superior overall survival rates than the poor responders group (*P*=0.044). In comparison, no significant difference was observed regarding overall survival and event-free survival rates when using the cutoff of 0.07 increment from ADC1 to ADC2. Regarding survival, the univariate analysis demonstrated that ADC change from MRI-1 to MRI-2 (ADC2−ADC1) (HR [hazard ratio] 2.43, *P*=0.57, 95% CI 0.11–52.71) was not a significant prognostic variable for overall survival, nor was tumour volume (HR 0.99, *P*=0.87, 95% CI 0.99–1.01). Conversely, metastases at diagnosis (HR 5.68, *P*=0.02, 95% CI 1.29–25.02) and poor histological response to chemotherapy (HR 5.00, *P*=0.05, 95% CI 1.00–25.00) were significant prognostic variables for overall survival. The multivariate analysis showed that initial metastases (HR 3.41, *P*=0.141, 95% CI 0.67-17.44) and poor histological response (HR 2.81, *P*=0.276, 95% CI 0.43–19.14) were not independently significant for overall survival.

## Discussion

In agreement with our previous results [16], the good responders demonstrated higher ADC values at mid-course neoadjuvant chemotherapy and a superior increase in ADC value from MRI-1 to MRI-2. However, the threshold of 0.07 increment in ADC value from baseline to the middle of treatment could only identify 25% of poor responders with 100% specificity with 65% accuracy, hence lower than the 57% sensitivity that we had previously observed [3, 17].

In addition, we were able to demonstrate that ADC values recorded at the end of chemotherapy were significantly correlated to the rate of necrosis at histology, which was not the case in our preliminary cohort, but is in line with previous studies [13, 18, 19].

Overall survival rates were significantly superior among the good histological responders compared to the poor responders, in keeping with current knowledge [3, 17]. On the other hand, the 0.07 increment cutoff from MRI-1 to MRI-2 could not establish any significant differences between survival rates of both groups, as defined by ADC measurement. We failed to demonstrate the increase in ADC from diagnosis to mid-course chemotherapy MRI to be a prognostic factor of survival, in comparison to reported high-risk factors, i.e. a poor histological response to chemotherapy and initial metastases [2, 4].

Thus, we have demonstrated the potential for DWI in distinguishing good from poor histological responders to neoadjuvant chemotherapy by assessing the differential in mean tumoural ADC values from diagnosis to mid-course MRI. We put forward a simple time-saving method of quantitative analysis of DWI that could readily be put into daily practice while reviewing images, to assist visual interpretation of conventional images and to aid in monitoring the efficiency of preoperative chemotherapy.

French management of long-bone osteosarcoma in adolescents and young adults is based on a methotrexate-etoposide-ifosfamide neoadjuvant regimen followed by limb-sparing surgery and postoperative chemotherapy [4, 20]. This combined treatment has improved the long-term survival of osteosarcoma patients. A plateau reached over the last decades may partly be explained by the late assignment of patients to high-risk groups. According to this protocol, early progression during the preoperative phase would lead to a modified treatment strategy, with a switch to doxorubicin-cisplatinum or early surgery. At present, one of the main predictive factors for local relapse and distant metastasis is the degree of cellular necrosis secondary to neoadjuvant chemotherapy [5–7]. The histological response is assessed by analysing the surgical specimen after completing 14 weeks of neoadjuvant treatment, excluding the delay incurred by fixation, decalcification and analysis of the pathological sample. Early detection of patients who respond poorly to preoperative chemotherapy may be an important issue in managing osteosarcoma in young patients.

Advances in understanding the basis of osteosarcoma pathogenesis call for alternative and earlier methods of identifying patients at higher risk of relapse who may benefit from novel molecular therapies [1, 21]. Thus, the major challenge for radiology is to find an early prognostic factor that will allow the neoadjuvant treatment regimen to be adjusted. There is sufficient evidence that visual interpretation of conventional MRI is no longer enough. Sole assessment of tumour volume change during neoadjuvant chemotherapy has limited correlation with prognosis because tumour shrinking mainly concerns the soft-tissue component while the intramedullary extension is spared [8–10, 22]. Functional imaging of tumour behaviour is therefore warranted.

DWI reflects tissue microstructure by detecting the free mobility of water molecules in tissues. Thus, it provides functional information on tumour composition, which can be assessed quantitatively by measuring the corresponding ADC map. For instance, hypercellular tumoural areas restrict free mobility of water and exhibit low ADC values, whereas necrotic areas secondary to chemotherapy-induced cellular lysis will exhibit higher ADC values [11, 23].

Several authors support the functional added value of DWI in evaluating the response to neoadjuvant chemotherapy of osteosarcoma [14, 15, 18, 19]. Wang and coauthors [15] brought a further understanding to behavioural differences between good and poor responding osteosarcoma by qualitative analysis of their ADC histogram distribution. A post-chemotherapy ADC histogram of good responders is flat, wide and asymmetrical with a shift to the right of the coordinate, reflecting higher intratumoural heterogeneity and higher values of ADC from necrosis, whereas poor responders retain a sharp and high ADC histogram over time, suggesting that there is no significant modification in tumoural composition. More recently, intravoxel incoherent motion technique has also been applied to demonstrate the increase in true molecular diffusion of osteosarcoma after neoadjuvant chemotherapy by isolating the perfusion effect from microvasculature, but again with measurements obtained preoperatively and compared to a different reference standard than histological response [24]. The main weakness of previous studies is that measurements obtained at the end of the neoadjuvant regimen are too close to surgery to allow any treatment optimization. Therefore, ADC measurements assessed earlier during the course of therapy should be considered.

An emerging and promising approach for the development of imaging biomarkers could rely on radiomics based on MRI. The mining of advanced quantitative features from osteosarcoma combined with clinical and genomic data in high-order statistics may be able to define predictive models for risk stratification of patients before initiating neoadjuvant treatment [25].

Our study has several limitations. First, it is monocentric with a small sample size with obvious impact on the statistical power of our results. This may explain part of the differences observed with our previous findings and our failure to demonstrate independency upon potential prognosticators through multivariate analysis. Although osteosarcoma is the most common bone tumour in adolescents and young adults, it is a relatively rare disease. To ensure the homogeneity of our series, we stopped enrolling patients in 2016 when our MR unit was changed to a higher field magnet (3 T) because of the dependency of DWI upon multiple parameters, including the magnetic field strength, manufacturer, pulse sequences, and post-processing in terms of signal-to-noise and quantification of ADC values [11, 26]. Despite these technical limitations, the implementation of our methodology still has to be validated by larger and prospective clinical studies. Second, we did not take into account the histological subtypes of conventional osteosarcoma because of our small sample size. Telangiectatic osteosarcomas were excluded from the study because of their essentially cystic matrix that could have distorted ADC values. Still, matrix differences between subtypes could account for the variability of our ADC measurements [27, 28]. In addition, we have chosen to measure the mean ADC value of the greatest tumoural surface that could be segmented on a coronal plane in order to evaluate as much osseous material in the same plane as the pathological slice, i.e. the reference standard. The corresponding MRI slice is thicker than the pathologist’s (20 mm and 5 mm, respectively) with possibly different representations of tumour histological modifications. We did not apply any signal-to-noise threshold to exclude uncertain low values resulting from noise because we did not benefit from a preprocessing algorithm that would control accuracy of our measurements. Automated solutions that allow preprocessing control for precision of voxel-based ADC values before quantitative extraction would be ideal, despite being beyond the scope of our study. The average ADC value may not accurately reflect intra- and intertumoural heterogeneity. Minimal ADC value could better indicate the higher cellular components of the tumour, purportedly enabling more precise longitudinal monitoring under neoadjuvant chemotherapy [18]. More advanced statistics could be applied on ADC data, such as skew and kurtosis of distribution, which may be even more representative of the rate of post-chemotherapy necrosis [15]. Finally, a more accurate apprehension of osteosarcoma physiopathology and outcome heterogeneity could be achieved by integrating quantitative DWI with other acquisition sequences and patient characteristics to produce future evidence-based decision-making tools [29].

## Conclusion

Our study provides further insights into the potential for mid-course chemotherapy DWI to serve as an early imaging tool that could have clinical impact on monitoring young patients with long-bone osteosarcoma. The analysis of functional information from diffusion should be reconsidered within the scope of current advances in radiomics applied to oncology.
